# Supplementary material for: DMRforPairs: identifying Differentially Methylated Regions between unique samples using array based methylation profiles
Source: BMC Bioinformatics. 2014 May 15;15:141. doi: 10.1186/1471-2105-15-141 (PMC4046028; doi:10.1186/1471-2105-15-141)
Supplement: Additional file 2 — DMRforPairs output for the comparison of A431-MCF7 and NA17018-NA17105. Please start from the HTML files in each folder. Available via the BMC Bioinformatics website. [file 1471-2105-15-141-S2.zip › 1394847754114233_MOESM2_ESM/A431_MCF7/figures/23928.pdf]

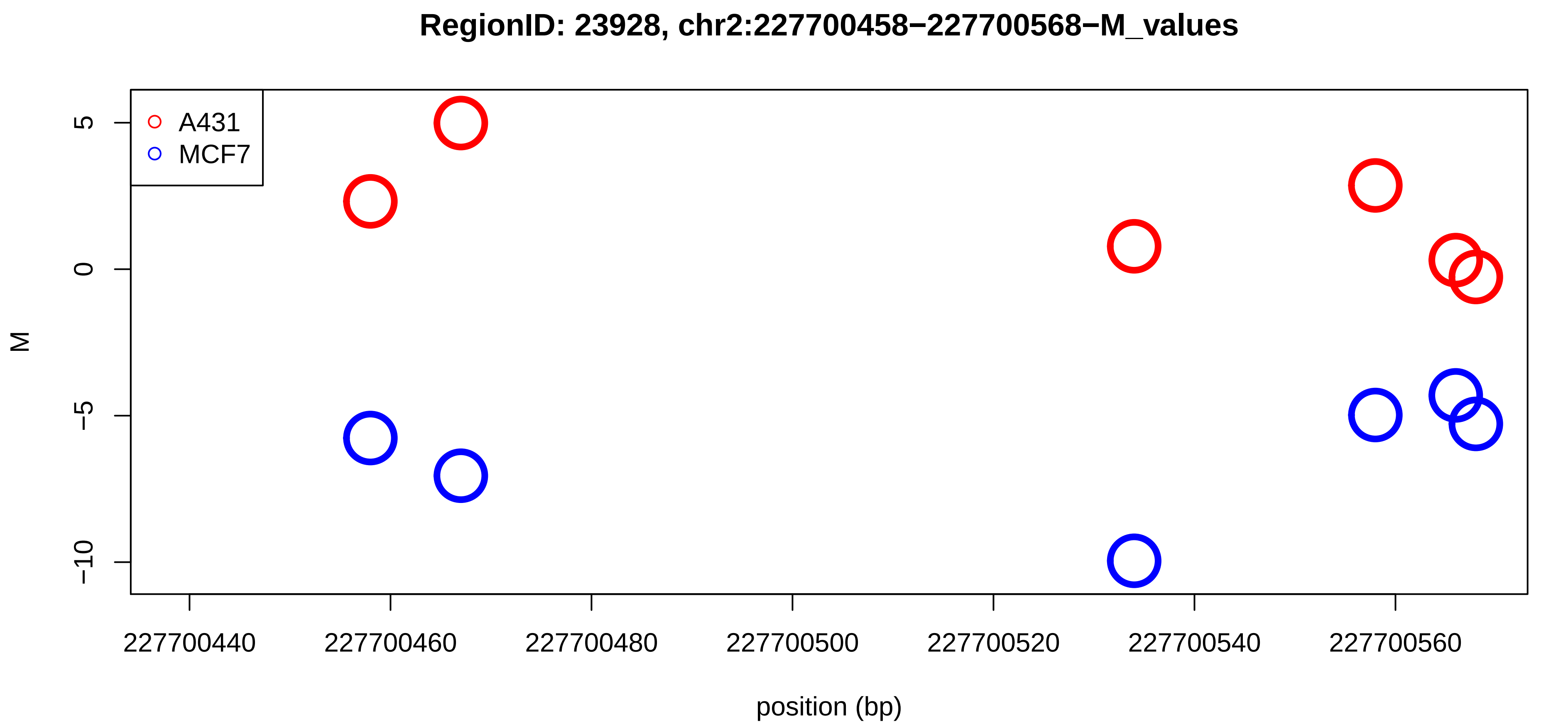
RegionID: 23928, chr2:227700458−227700568−M_values
A431
MCF7
M
5
0
−5
−10
227700440
227700460
227700480
227700500
227700520
227700540
227700560
position (bp)

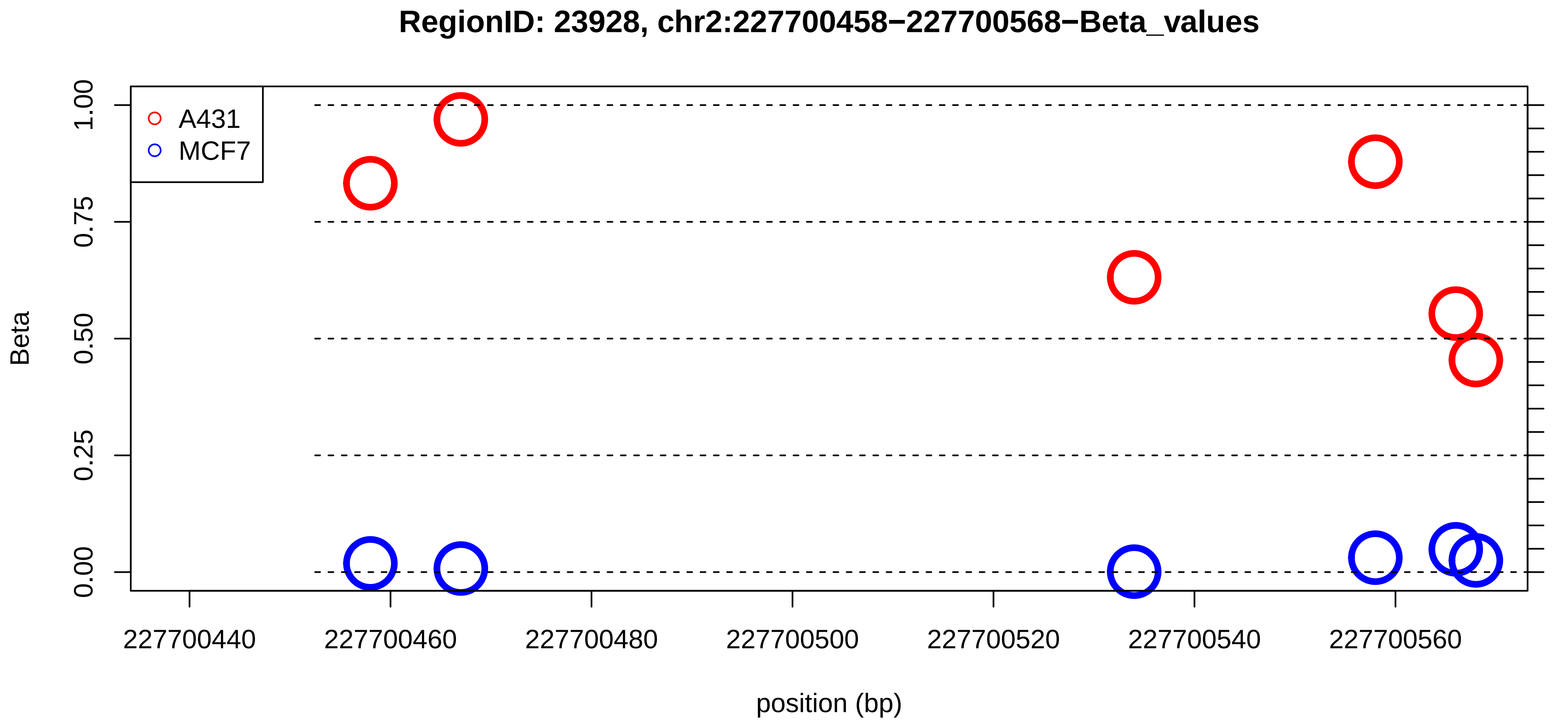
RegionID: 23928, chr2:227700458−227700568−Beta_values
A431
MCF7
Beta
1.00
0.75
0.50
0.25
0.00
227700440
227700460
227700480
227700500
227700520
227700540
227700560
position (bp)

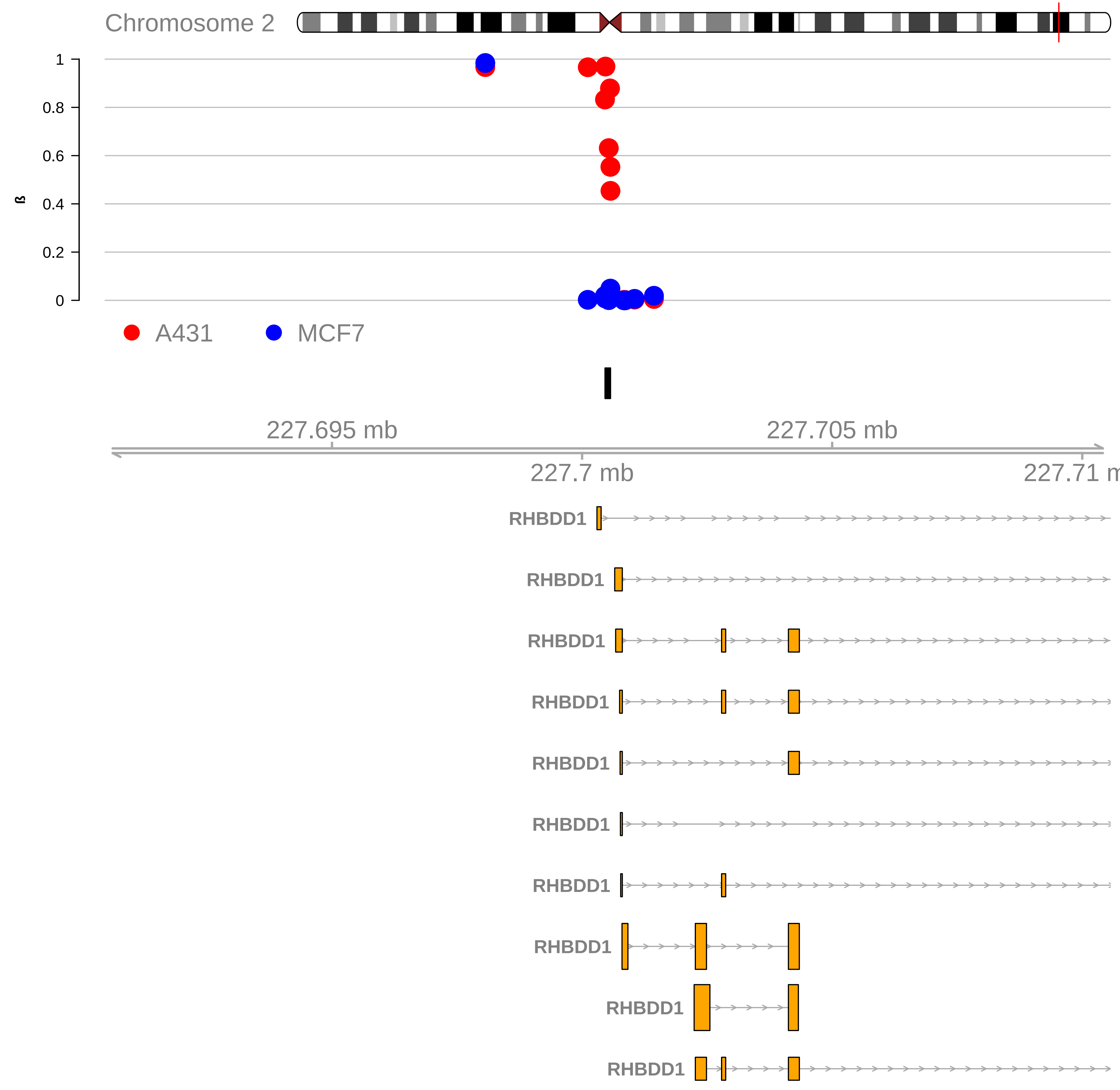
Chromosome 2
1
0.8
0.6
0.4
0.2
0
β
A431
MCF7
227.695 mb
227.705 mb
227.7 mb
227.71 m
RHBDD1
RHBDD1
RHBDD1
RHBDD1
RHBDD1
RHBDD1
RHBDD1
RHBDD1
RHBDD1
RHBDD1
